# RNA interference targeting the platelet-derived growth factor receptor β subunit ameliorates experimental hepatic fibrosis in rats

**DOI:** 10.1111/j.1478-3231.2008.01759.x

**Published:** 2008-11

**Authors:** Si-Wen Chen, Xing-Rong Zhang, Chong-Ze Wang, Wei-Zhong Chen, Wei-Fen Xie, Yue-Xiang Chen

**Affiliations:** 1 Department of Gastroenterology, Changzheng Hospital, Second Military Medical UniversityShanghai, China; 2 Department of Geriatrics, Renji Hospital, Shanghai Jiaotong University School of MedicineShanghai, China

**Keywords:** cell proliferation, gene therapy, hepatic fibrosis, hepatic stellate cells, platelet-derived growth factor receptor β subunit (PDGFR-β), RNA interference

## Abstract

**Background/Aims:**

Platelet-derived growth factor (PDGF) is the strongest stimulator of the proliferation of hepatic stellate cells (HSCs). PDGF receptor β subunit (PDGFR-β) is acquired on HSCs proliferation induced by PDGF. In this study, we aim to investigate the effect of PDGFR-β small interference RNA (siRNA) on experimental hepatic fibrosis.

**Methods:**

We constructed a PDGFR-β siRNA expression plasmid and investigated its effect on the activation of HSCs. Bromodeoxyuridine incorporation was performed to investigate the effect of PDGFR-β siRNA on HSCs proliferation. A hydrodynamics-based transfection method was used to deliver PDGFR-β siRNA to rats with hepatic fibrosis. The distribution of transgenes in the liver was observed by immunofluorescence. The antifibrogenic effect of PDGFR-β siRNA was investigated pathologically.

**Results:**

Platelet-derived growth factor receptor-β subunit siRNA could significantly downregulate PDGFR-β expression, suppress HSCs activation, block the mitogen-activated protein kinase signalling pathway and inhibit HSCs proliferation *in vitro*. PDGFR-β siRNA expression plasmid could be delivered into activated HSCs by the hydrodynamics-based transfection method, and remarkably improve the liver function of the rat model induced by dimethylnitrosamine and bile duct ligation. Furthermore, the progression of fibrosis in the liver was significantly suppressed by PDGFR-β siRNA in both animal models.

**Conclusions:**

Platelet-derived growth factor receptor-β subunit siRNA may be presented as an effective antifibrogenic gene therapeutic method for hepatic fibrosis.

Hepatic fibrosis characterized by the excess production and deposition of extracellular matrix (ECM) components is the common final pathway in most chronic liver diseases. Although the exact mechanisms of hepatic fibrosis are not fully understood, it is known that the activation of hepatic stellate cells (HSCs) characterized by the morphological transition to myofibroblast-like cells is the key event of the pathogenesis of hepatic fibrosis (1, 2). The phenotype of activated HSCs may be influenced by several growth factors and cytokines, including the platelet-derived growth factor (PDGF), which is the most potent mitogen for activated HSCs. In response to PDGF, activated HSCs display enhanced mitogenicity and increased production of ECM (3).

The family of PDGF contains four polypeptide chains: PDGF-A, -B, -C and - D. PDGF-A and -B are secreted as homodimers or heterodimers and are involved in autocrine and paracrine stimulation of HSCs proliferation (4), while the latter two form only homodimers (5–7). The PDGF receptor is a structurally related tyrosine kinase that is a homodimer or a heterodimer of α or β subunits. The PDGF receptor (PDGFR)-α subunit is constitutively expressed in quiescent HSCs, whereas PDGFR-β is acquired when, during the activation of HSCs, PDGF-A binds to the α subunit only, while PDGF-B is a ligand for both the receptor subunits (4, 8). It is well known that PDGF-BB is the strongest stimulator of HSCs proliferation. The expression of PDGF and PDGFR-β was demonstrated to correlate with necrotic inflammation and fibrosis in fibrotic liver, clearly reflecting the essential role of PDGF and PDGFR-β in the pathogenesis of hepatic fibrosis (9). Therefore, a number of studies have focused on the treatment of hepatic fibrosis by targeting PDGF, PDGFR and molecules related to the PDGF intracellular signalling transduction pathway, and have achieved a favourable antifibrogenic effect. In this study, we constructed a PDGFR-β short hairpin RNA (shRNA) expression vector, which showed high gene-silencing efficacy and an antiproliferative effect *in vitro*, and investigated its antifibrogenic effect on experimental hepatic fibrosis induced by dimethylnitrosamine (DMN) and bile duct ligation (BDL).

## Materials and methods

### Generation of plasmids

The 21-nucleotide small interference RNA (siRNA) against rat PDGFR-β gene with a high gene-silencing efficacy (sense: GUGGACUCCGAUACUUACUtt; antisense: AGUAAGUAUCGGAGUCCACtt) and its negative control RNA (sense: UUCUCCGAACGUGUCACGUtt; antisense: ACGUGACACGUUCGGAGAAtt) were chemically synthesized by Shanghai Sangon Biological Engineering Technology & Service Co. Ltd (Shanghai, China). The shRNAs corresponding to PDGFR-β siRNA and its negative control containing the 9-nucleotide loop sequence (TTCAAGAGA) flanked by sense and antisense siRNA sequences were synthesized by the polymerase chain reaction (PCR). The PDGFR-β shRNA was inserted immediately into the miR30-based shRNA expression system of the pCMR30 vector (Genechem, Shanghai, China) to generate a pCMV–shRNA–red fluorescent protein (RFP), in which PDGFR-β shRNA was driven by the CMV promoter and RFP was the reporter gene. To generate pCMV–shRNA–LacZ, the fragment containing miR30-based PDGFR-β shRNA was PCR-cloned into the HindIII site of the pMIR-REPORT β-gal reporter control vector (Ambion, Austin, TX, USA), which supplies the β-gal reporter gene. As a negative control, we generated pCMV–NC–LacZ with miR30-based PDGFR-β shRNA in pCMV–shRNA–LacZ replaced by negative control RNA. All the generated plasmids were identified by PCR and sequencing. Detailed vector maps and sequence information are available on request.

### Cell culture and transfection

The rat HSC-T6 cell line was a generous gift from Dr Scott Friedman of the Mount Sinai School of Medicine in New York, USA (10). The cells were cultured in Dulbecco's modified medium containing 10% fetal bovine serum (Gibco, Grand Island, NY, USA). Transient transfection using Lipofectamine 2000 (Invitrogen, Carlsbad, CA, USA) was carried out in six-well plates with 6 μg plasmids/35 mm culture plate when cells reach 60–70% confluence. The medium for culture was changed with fresh medium at 6 h after transfection. The serum content was reduced to 0.25% fetal bovine serum 24 h later and the cells were stimulated with 20 ng/ml PDGF-BB (Sigma, Saint Louis, MO, USA) for another 24 h.

### Reverse transcription-polymerase chain reaction assay

Total RNA was isolated with Trizol reagent (Invitrogen) from the cells that were transfected with plasmids and/or stimulated by PDGF-BB. Reverse transcription-polymerase chain reaction (RT-PCR) was performed using the RT-PCR kit (Takara, Otsu, Japan). Primers against PDGFR-β mRNA (sense: CACCATTTCGAGCACCTT TGT; antisense: AGGGCACTCCGAAGAGGTAA), β-actin mRNA (sense: CTTGACCCTGAAGTATCCC; antisense: TATTGAAGTGGTGGTGTCG) and α-smooth muscle actin (α-SMA) mRNA (sense: CAGGGAGTGAT GGTTGGA; antisense: AGACGGAGTACGGTAGTA) were designed according to Pubmed GenBank and synthesized by Shanghai Sangon Biological Engineering Technology & Service Co. Ltd (China). Amplification of β-actin was used for the determination of the internal mRNA expression levels. Briefly, PCR was performed at 95 °C with initial denaturation for 5 min, followed by 30 cycles of amplification at 94 °C (1 min), 59 °C (1 min) and 72 °C (1 min). Finally, the samples were extended at 72 °C for 10 min. The specificity of amplification was checked by assessing whether a fragment of the expected size had been obtained with the positive control. Aliquots of the synthesized PCR products were separated by electrophoresis on a 1.5% agarose gel and analysed by gel-pro 3.1 software.

### Western blot analysis

The cells (being transfected with plasmids and/or stimulated by PDGF-BB) and the frozen liver tissues collected in the animal experiments were homogenized in lysis buffer. Phosphatase inhibitor (Sigma) was added before use to prevent protein dephosphorylation. Protein lysates were sonicated and centrifuged at 10 000 r.p.m. (13 428 *g*) at 4 °C for 10 min. Western blot was performed as described elsewhere (11). The primary antibodies used for Western blot were PDGFR-β polyclonal antibody (Santa Cruz; Santa Cruz, CA, USA), α-SMA polyclonal antibody (Abcam, Cambridge, UK), β-actin polyclonal antibody (Chemicon, Billerica, MA, USA), extracellular-signal-related kinase (ERK) and pERK polyclonal antibody (Santa Cruz). The bands were visualized using the ECL Plus reagents (Sigma) according to the manufacturer's instructions. Protein signals were normalized to β-actin levels.

### Proliferation assay

After plasmid transfection and/or PDGF-BB stimulation, cells were stimulated with 10 ìM 5-bromodeoxyuridine (BrdU; Upstate, Billerica) for 1 h. For immunofluorescence analysis, cells were fixed with 4% paraformaldehyde solution and then incubated with HCl and borate buffer. BrdU in the nucleus was detected by immunocytochemical staining using a mouse monoclonal fluorescein isothiocyanate (FITC)-labelled antibody to BrdU (Abcam). Light nuclear counterstaining was performed with 4,6-diamidino-2-phenylindole (Roche, Nutley, NJ, USA) before mounting. Cells were analysed using a Leica TCS SP2 confocal microscope system (Leica, Solms, Germany). HSCs proliferation was measured by green staining of nuclei that had incorporated BrdU. Results were expressed as mean±SD of cells that had incorporated BrdU (percentage of BrdU-positive cells). The flow cytometry analysis of BrdU incorporation was performed according to the protocol of BD Biosciences (http://www.bdbiosciences.com/pharmingen/protocols/BrdU_Incorporation.shtml) and analysed using a FACS Aria Flow Cytometer System (BD Biosciences, San Jose, CA, USA).

### Localization of LacZ gene expression in bile duct ligation and dimethylnitrosamine models

All the animals [Sprague–Dawley (SD) rats] weighing about 200 g were housed in cages with stainless-steel wire tops and with 12-h light–dark cycles under standard animal laboratory conditions in the specific pathogen-free-grade animal room of the Experimental Animal Center of the Second Military Medical University. The rats had free access to standard rat chow and water. This study was approved by the Local Ethical Committee of the Second Military Medical University.

To induce the DMN model, 10 male SD rats received DMN (Tianjin Chemical Reagent Research Institute, Tianjin, China) dissolved in saline at a dose of 10 μl DMN/kg by a peritoneal injection for three consecutive days each week for 3 weeks. On the 21st day, three survivors were treated with pCMV–NC–LacZ by the hydrodynamics-based transfection method. Briefly, a total of 15 ml Ringer's solution containing 100 μg pCMV–NC–LacZ/200 g body weight was injected in 17 s via the tail vein. Another three surviving DMN-treated rats without plasmid transfection were used as control. For the BDL model, 10 male SD rats were anaesthetized with chloral hydrate, and the common bile duct was double ligated with 4-0 silk ligatures and transected. On the 14th day, pCMV–NC–LacZ treatment with the hydrodynamics-based transfection method was performed in three survivors as described above and another three surviving BDL rats were used as untransfected control.

Twenty-four hours after pCMV–NC–LacZ treatment, all the BDL- and DMN-treated rats were anaesthetized and perfused, the livers were taken out and post-fixed with 4% paraformaldehyde and 0.2% picric acid in phosphate buffer and cut into 8-μm-thick sections. The sections were incubated with rabbit anti-α-SMA polyclonal antibody (Bios, Beijing, China) and mouse anti-β-gal monoclonal antibody (Promega, Madison, WI, USA) for 24 h at 4 °C, then washed in phosphate-buffered saline (PBS) and incubated with FITC-labelled anti-rabbit immunoglobulin G (IgG) antiserum and Cy5-labelled anti-mouse IgG antiserum (Jackson, West Grove, PA, USA) for 30 min at 37 °C. The sections were again washed in PBS and then transferred to slides and coverslipped. α-SMA and β-gal immunofluorescence were visualized in the same sections using a Leica TCS SP2 confocal microscope system (Leica).

### Animal experiments of gene therapy

In the DMN model, 48 male SD rats weighing about 200 g were randomly divided into the normal group (*n*=12) and the DMN-treatment group (*n*=36). All the animals in the DMN-treatment group received DMN treatment as described above, and were randomly assigned to the following three groups (12 in each): pCMV–shRNA–LacZ treatment group, Ringer's solution group and pCMV–NC–LacZ treatment as the negative control group.

For the BDL model, 55 male SD rats weighing about 200 g were randomly assigned to the normal group (*n*=11), the sham-operated group (*n*=11) and the BDL group (*n*=33). In the BDL group, hepatic fibrosis was induced as described above. In sham-operated rats, blunt dissection, but without ligation and transection of the common bile duct, was performed. The 33 BDL rats were divided into three groups as for the DMN model.

All the solutions were injected by a hydrodynamics-based transfection method. Briefly, a total of 15 ml solution containing 100 μg plasmids/200 g body weight was injected in 17 s via the tail vein at 24 h before DMN treatment or BDL operation, and then 10 ml containing 100 μg plasmids/200 g body weight in 17 s via the tail vein every 5 days for 3 weeks in the DMN model or 2 weeks in the BDL model. At the end of 2 or 3 weeks, the rats were sacrificed and serum samples were collected for biochemical tests. The right lobe of the livers was taken from the abdominal cavity, then soaked with 10% neutral formaldehyde for histology and preserved at −70 °C for Western blot analysis.

### Evaluation of hepatic fibrosis

The pathogenesis of hepatic fibrosis induced by DMN or BDL and the effects of PDGFR-β shRNA were evaluated through Masson's trichrome staining. The stained sections were examined under an Olympus research microscope, BX61 (Olympus, Tokyo, Japan). The stage of fibrosis was graded as stages 0 (no fibrosis) to 4 (cirrhosis), as shown in Table 1. The densitometry of the collagen was quantified using image-pro plus software.

**Table 1:** Stages of fibrosis

Description	Score	Definition
No fibrosis	0	Normal connective tissue
Portal fibrosis	1	Fibrous portal expansion
Periportal fibrosis	2	Periportal fibrosis with short septa extending into lobules or rare porto-portal septa (intact architecture)
Septal fibrosis	3	Fibrous septa reaching adjacent portal tracts and terminal hepatic venule (architecture distortion, but no obvious cirrhosis)
Cirrhosis	4	Diffuse nodular formation

### Immunohistochemistry for α-smooth muscle actin and platelet-derived growth factor β subunit on liver sections

The paraffin sections were deparaffinized using xylene and alcohol and hydrated with water, and then treated with PDGFR-β polyclonal antibody or α-SMA monoclonal antibody (Boster, Wuhan, China) as primary antibodies, followed by biotinylated secondary antibody and streptavidin peroxidase. The sections were counterstained with Mayer's haematoxylin, treated with ammonia water and mounted using an aqueous-based mounting medium and photographed. The staining intensity of α-SMA and PDGFR-β was quantified using image-pro plus software.

### Statistics

Results were presented as means of three independent experiments (±SD). In the semiquantitative analysis of histological staging, nonparametric tests (Wilcoxon's test) were used; other statistical analyses were performed using an unpaired Student's *t* test. Differences were considered to be significant or highly significant at *P*<0.05 or *P*<0.01 respectively.

## Results

### Platelet-derived growth factor β subunit short hairpin RNA downregulated platelet-derived growth factor β subunit expression and suppressed hepatic stellate cells activation *in vitro*

**Fig. 1:**
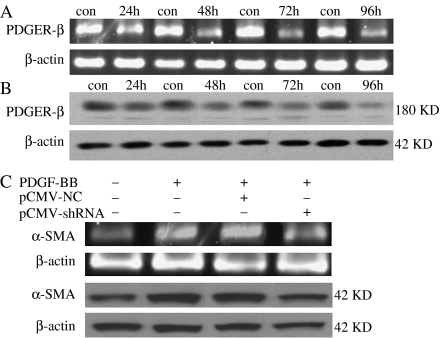
Effect of platelet-derived growth factor β subunit (PDGFR-β) short hairpin RNA (shRNA) on PDGFR-β and α-smooth muscle actin (α-SMA) expression in hepatic stellate cells (HSCs). (A) and (B) show the PDGFR-β mRNA and protein expression after transfection with PDGFR-β shRNA for 24, 48, 72 and 96 h. ‘con’ represents HSCs without treatment. (C) Shows α-SMA mRNA and protein expression in HSCs without treatment, HSCs with platelet-derived growth factor (PDGF)-BB, HSCs with pCMV–NC–LacZ and PDGF-BB and HSCs with pCMV–shRNA–LacZ and PDGF-BB. The results demonstrated that the α-SMA expression of HSCs was significantly increased (*P=*0.002) after PDGF-BB stimulation, which was impeded by PDGFR-β shRNA transfection (*P=*0.001). pCMV–shRNA, plasmid pCMV–shRNA—LacZ; pCMV–NC, negative control of plasmid pCMV–NC–LacZ.

Reverse transcription-polymerase chain reaction results showed that PDGFR-β shRNA downregulated the PDGFR-β mRNA expression by 40±3.14, 55±4.67, 72±4.20 and 55±6.19% at 24, 48, 72 and 96 h respectively (Fig. 1A). In addition, PDGFR-β protein expression was downregulated by 33±4.51, 47±5.72, 60±7.59 and 82±7.13% at 24, 48, 72 and 96 h respectively, indicating that the highest gene-silencing efficacy of PDGFR-β shRNA appeared in 72 h, while the protein level was reduced more significantly in 96 than 72 h, which may be because of the time period of transcription and translation from mRNA to protein (Fig. 1B). As the activation of HSCs characterized by the expression of α-SMA is the key event of the pathogenesis of hepatic fibrosis, the effects of PDGFR-β shRNA on the mRNA and protein expression of α-SMA were investigated in HSCs. The results revealed that the α-SMA expression in HSCs had increased significantly after PDGF-BB stimulation, which was impeded by PDGFR-β shRNA transfection (Fig. 1C).

### Platelet-derived growth factor β subunit short hairpin RNA blocked the mitogen-activated protein kinase signalling transduction pathway and inhibited hepatic stellate cells proliferation *in vitro*

**Fig. 2:**
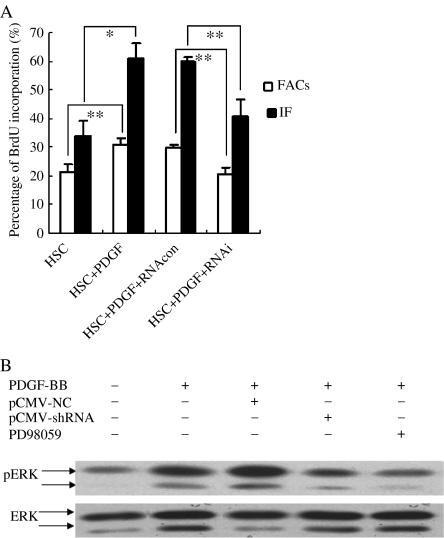
Effect of platelet-derived growth factor β subunit (PDGFR-β) short hairpin RNA (shRNA) on the proliferation of hepatic stellate cells (HSCs). (A) showed statistical results of bromodeoxyuridine incorporation detected by immunofluorescence (IF) and flow cytometry (FACs) in HSCs without treatment (HSC), HSCs with platelet-derived growth factor (PDGF)-BB (HSC+PDGF), HSCs with pCMV–NC–LacZ and PDGF-BB (HSC+PDGF+RNA_con_) and HSCs with pCMV–shRNA–LacZ and PDGF-BB (HSC+PDGF+RNA interference). The results revealed that PDGF-BB significantly promoted the proliferation of HSCs (*P=*0.008 with flow cytometry analysis and *P=*0.012 with immunofluorescence), which was inhibited by pCMV–shRNA–LacZ (*P=*0.003 with flow cytometry analysis and *P=*0.006 with immunofluorescence). ^*^P<0.05, ^**^P<0.01. (B) shows the representative graph of extracellular-signal-related kinase (ERK) phosphorylation in HSCs without treatment, HSCs with PDGF-BB, HSCs with pCMV–NC–LacZ and PDGF-BB, HSCs with pCMV–shRNA–LacZ and PDGF-BB and HSCs with PD98059 and PDGF-BB by Western blot analysis. PD98059 is a specific inhibitor of ERK phosphorylation.

To investigate the effect of PDGFR-β shRNA on HSCs proliferation, BrdU incorporation was used and analysed by immunofluorescence and flow cytometry. The results showed that the HSCs proliferation promoted by PDGF-BB stimulation was significantly suppressed by PDGFR-β shRNA (Fig. 2A). Furthermore, Western blot analysis revealed that the phosphorylation of ERK had increased significantly after stimulation by PDGF-BB, which was impeded by PDGFR-β shRNA or PD98059 (Fig. 2B). As a positive control, PD98059 is a specific inhibitor of ERK phosphorylation.

### Platelet-derived growth factor β subunit short hairpin RNA could be delivered into activated hepatic stellate cells in rats

**Fig. 3:**
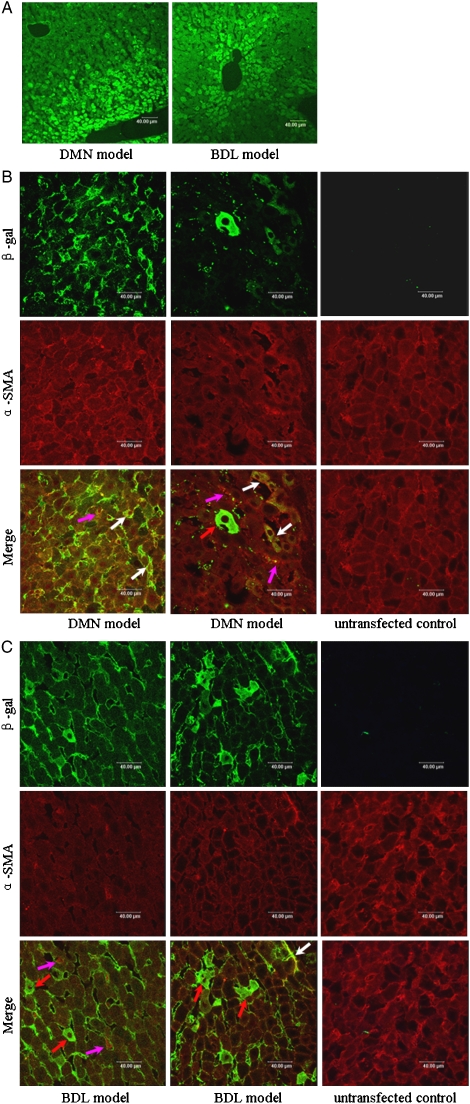
Localization of LacZ gene expression in the hepatic fibrosis rat model. The immunofluorescence graph of β-gal staining (green) in a dimethylnitrosamine (DMN)-treated rat and a bile duct ligation (BDL) rat (A) showed that the transgenes were widely distributed in hepatic lobules, mainly periportal and pericentral areas, with a magnification of × 40. The double-label immunofluorescence graph of β-gal (green) and α-smooth muscle actin (α-SMA) (red) showed that in the liver section of DMN-treated rats (B) and BDL rats (C), the transgenes could be expressed in α-SMA-positive HSCs, magnification of × 63. The ‘untransfected control’ rats, which indicate DMN-treated rats (B) or BDL rats (C) without pCMV–short hairpin RNA (shRNA)–LacZ transfection, were used to exclude unspecific staining of β-gal. The white arrows point to the cells with α-SMA and β-gal, double positive, the red arrows point to the hepatocytes residing in the liver cell cord and the pink arrows point to the α-SMA-positive HSCs that were not transfected with pCMV–shRNA–LacZ. The results illustrated that pCMV–shRNA–LacZ could be delivered into activated HSCs by a hydrodynamics-based transfection method.

Because PDGFR-β shRNA was confirmed to suppress HSCs activation and inhibit HSCs proliferation *in vitro*, what concerned us was whether it could be delivered into activated HSCs by a hydrodynamics-based transfection method *in vivo*. The double-label immunofluorescence showed that pCMV–shRNA–LacZ could be delivered into liver cells, including hepatocytes and α-SMA-positive activated HSCs in DMN rats and BDL rats, successfully by the hydrodynamics-based transfection method (Fig. 3).

### Platelet-derived growth factor β subunit short hairpin RNA relieved the liver injury in rats

**Fig. 4:**
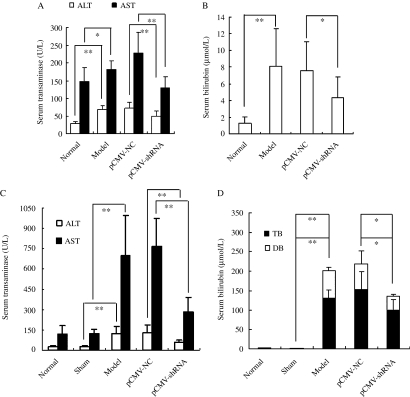
Effect of platelet-derived growth factor β subunit (PDGFR-β) short hairpin RNA (shRNA) on serum biochemical parameters of experimental hepatic fibrosis induced by dimethylnitrosamine (DMN) or bile duct ligation (BDL) treatment. (A) and (B) show the effect of PDGFR-β shRNA on the serum alanine aminotransferase (ALT), aspartate aminotransferase (AST) and total bilirubin (TB) levels of experimental hepatic fibrosis induced by DMN. The results demonstrated that serum TB, ALT and AST levels were significantly increased in the DMN model group compared with the normal group (TB: *P*=0.001; ALT: *P*<0.001; AST: *P*=0.044), which was impeded by pCMV–shRNA–LacZ treatment (TB: *P*=0.036; ALT: *P*=0.008; AST: *P*<0.001 compared with the pCMV–NC–LacZ treatment group); (C) and (D) show the effect of PDGFR-β shRNA on the serum TB, direct bilirubin (DB), ALT and AST levels of experimental hepatic fibrosis induced by BDL. The results revealed that serum TB, DB, ALT and AST levels were increased significantly in the BDL model group compared with the sham-operated group (TB: *P*<0.001; DB: *P*<0.001; ALT: *P*=0.002; AST: *P*=0.001), which was inhibited by pCMV–shRNA–LacZ treatment (TB: *P*=0.048; DB: *P*=0.044; ALT: *P*=0.008; AST: *P*<0.001 compared with the pCMV–NC–LacZ treatment group). ^*^P<0.05, ^**^P<0.01.

Because PDGF and PDGFR-β play important roles in the pathogenesis of hepatic fibrosis, we investigated the effect of PDGFR-β shRNA on liver injury in rats. In the DMN animal model, the results revealed that serum total bilirubin (TB), alanine aminotransferase (ALT) and aspartate aminotransferase (AST) levels were significantly increased in DMN control rats compared with the normal rats, and significantly reduced in the pCMV–shRNA–LacZ treatment rats compared with the pCMV–NC–LacZ treatment rats (Fig. 4A and B). In the BDL animal model, the results demonstrated that serum TB, direct bilirubin, ALT and AST levels were significantly increased in the BDL control group compared with the sham-operated group, which was eliminated by pCMV–shRNA–LacZ treatment (Fig. 4C and D).

### Platelet-derived growth factor β subunit short hairpin RNA downregulated platelet-derived growth factor β subunit expression and inhibited hepatic stellate cells activation in rats

**Fig. 5:**
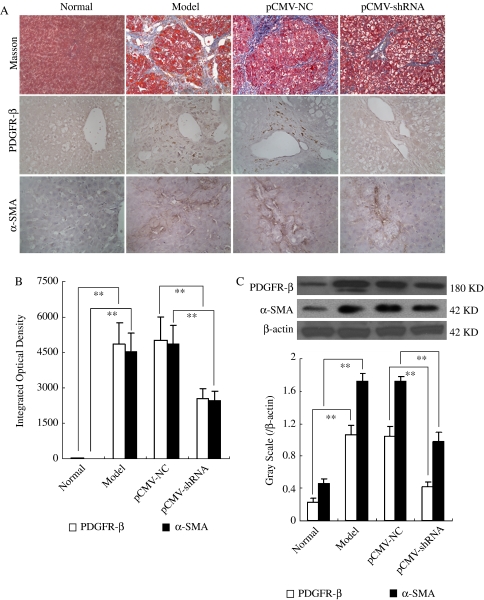
Effect of platelet-derived growth factor β subunit (PDGFR-β) short hairpin RNA (shRNA) on the histopathology and immunohistochemistry of experimental hepatic fibrosis induced by dimethylnitrosamine (DMN) treatment. (A) shows a representative graph of Masson's trichrome staining (the first line, magnification of × 20), PDGFR-β immunohistochemical staining (the second line, × magnification of 40) and α-smooth muscle actin (α-SMA) immunohistochemical staining (the third line, magnification of × 40). (B) shows the statistical results of PDGFR-β immunohistochemical staining and α-SMA immunohistochemical staining. (C) shows representative graphs of PDGFR-β and α-SMA protein expression by Western blot analysis (up) and the statistical results (below). ‘Normal’ represents the normal group, ‘Model’ represents the model control group, ‘pCMV–NC’ represents the pCMV–NC–LacZ treatment group and ‘pCMV–shRNA’ represents the pCMV–shRNA–LacZ treatment group. Results showed periportal fibrosis with fibrous septa reaching adjacent portal tracts and terminal hepatic venule, massive vacuolar degeneration of hepatocytes and intense neutrophilic infiltration in model control rats, while the hepatic fibrosis was relieved by pCMV–shRNA–LacZ treatment. PDGFR-β and α-SMA protein expressions were significantly increased in model control rats, which was impeded by pCMV–shRNA–LacZ treatment. ^**^P<0.01.

**Fig. 6:**
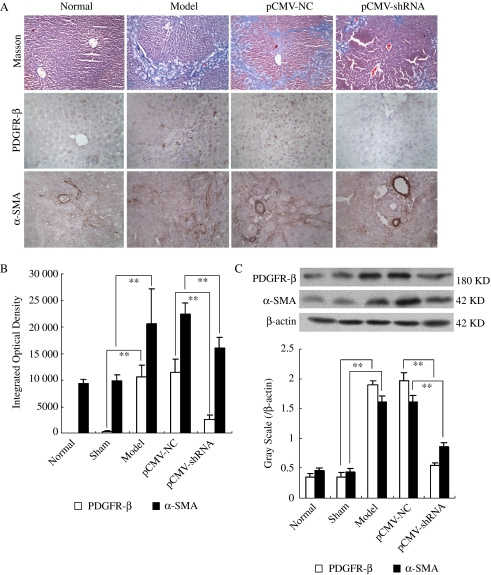
Effect of platelet-derived growth factor β subunit (PDGFR-β) short hairpin RNA (shRNA) on the histopathology and immunohistochemistry of experimental hepatic fibrosis induced by bile duct ligation (BDL). (A) Shows a representative graph of Masson's trichrome staining (the first line, magnification of × 20), PDGFR-β immunohistochemical staining (the second line, magnification of × 40) and α-smooth muscle actin (α-SMA) immunohistochemical staining (the third line, magnification of × 40). (B) Shows statistical results of PDGFR-β immunohistochemical staining and α-SMA immunohistochemical staining. (C) Shows representative graphs of PDGFR-β and α-SMA protein expression by Western blot analysis (up) and the statistical results (below). ‘Normal’ represents the normal group, ‘Sham’ represents the sham-operated group, ‘Model’ represents the model control group, ‘pCMV–NC’ represents the pCMV–NC–LacZ treatment group and ‘pCMV–shRNA’ represents the pCMV–shRNA–LacZ treatment group. Results show periportal fibrosis with short septa extending into lobules or porto–portal septa, severe cholestasis and bile duct hyperplasia in model control rats, while the hepatic fibrosis and cholestasis were all relieved by pCMV–shRNA–LacZ treatment. PDGFR-β and α-SMA protein expressions were significantly increased in model control rats, which was inhibited by pCMV–shRNA–LacZ treatment. ^**^P<0.01.

In the previous study, we have demonstrated that PDGFR-β shRNA could decrease the mRNA and protein expression of PDGFR-β and α-SMA *in vitro*. To further confirm its effect on the expression of PDGFR-β and α-SMA *in vivo*, a PDGFR-β shRNA expression plasmid was constructed and delivered into a rat model of hepatic fibrosis induced by DMN and BDL. In this study, Western blot analysis indicated that both PDGFR-β and α-SMA protein levels were significantly increased in DMN treatment rats and BDL rats, and remarkably reduced by pCMV–shRNA–LacZ treatment. The results were confirmed by immunohistochemical examination, implying that PDGFR-β shRNA could downregulate PDGFR-β expression and inhibit HSCs activation *in vivo* as well (Figs 5B, C and 6B, C).

### Platelet-derived growth factor β subunit short hairpin RNA ameliorated hepatic fibrosis in rats

Masson's trichrome staining of liver sections showed periportal fibrosis with fibrous septa extending to adjacent portal tracts and the terminal hepatic venule in BDL rats and DMN treatment rats. Severe cholestasis and bile duct hyperplasia were observed in BDL rats, and massive vacuolar degeneration of hepatocytes and intense neutrophilic infiltration were observed in DMN treatment rats, suggesting that the hepatic fibrosis animal model was established successfully. Hepatic fibrosis and cholestasis were significantly relieved by pCMV–shRNA–LacZ treatment in the DMN model or the BDL model compared with the pCMV–NC–LacZ treatment group, which was characterized as short septa extending into lobules or portal–portal septa (Figs 5A and 6A; Tables 2 and 3). The densitometry measurement showed that the percentage of collagen area was increased more than 80- or 60-fold in the DMN rats or the BDL rats compared with the normal rats (*P*<0.001 or *P*<0.001), while in pCMV–shRNA–LacZ treatment rats, it was cut down by about 45 or 60% compared with pCMV–NC–LacZ treatment rats in the DMN model or the BDL model (*P*=0.021 or 0.006). These results clearly indicated that PDGFR-β shRNA could ameliorate the hepatic fibrosis induced by BDL and DMN.

**Table 3:** Effect of PDGF receptor β subunit short hairpin RNA on hepatic fibrosis of bile duct ligation rats

Group/Stage	0–1	1–2	2–3	3–4	*U*
Normal	11	0	0	0	**–**
Sham	11	0	0	0	**–**
Model	0	0	8	1	4.0390**
pCMV-NC	0	0	6	2	**–**
pCMV-shRNA	0	8	0	0	3.4668##

To score the stage of fibrosis, three fields of microscope in the liver section of each rat were scored, and the average score was the stage of fibrosis.

** *P*<0.01 compared with the normal group.

## *P*<0.01 compared with the pCMV-NC-LacZ treatment group.

**Table 2:** Effect of PDGF receptor β subunit short hairpin RNA on hepatic fibrosis of dimethylnitrosamine rats

Group/Stage	0–1	1–2	2–3	3–4	*U*
Normal	12	0	0	0	–
Model	0	0	10	0	4.3875**
pCMV-NC	0	0	9	0	–
pCMV-shRNA	0	7	2	0	3.9622##

To score the stage of fibrosis, three fields of microscope in the liver section of each rat were scored, and the average score was the stage of fibrosis.

** *P*<0.01 compared with the normal group.

## *P*<0.01 compared with the pCMV-NC-LacZ treatment.

## Discussion

Hepatic fibrosis and its end-stage sequelae cirrhosis represent a major worldwide health problem. Considerable evidence suggests that HSCs activation is central to the fibrotic process. During hepatic fibrosis, HSCs undergo a process of activation, which coincides with increased cell proliferation and excessive production of ECM. An early proliferative response in HSCs is mainly mediated by PDGF and coupled with sequential expression of PDGFR-β. PDGF binding to PDGFR-β induces PDGFR autophosphorylation of tyrosine residues, initiating a mitogen-activated protein kinase (MAPK) signalling transduction pathway (4), which is necessary for the proliferation of activated HSCs induced by PDGF (12). Because PDGF and PDGFR-β have been implicated in hepatic fibrosis, many antifibrosis strategies focus on reducing the secretion of PDGF, inhibiting the coupling of PDGF and PDGFR, blocking the MAPK signal transduction pathway to reduce PDGF-induced HSCs proliferation subsequently. The antisense mRNA of the PDGF-B chain, tyrosine kinase inhibitor genistein and STI-571, selective Na^+^/H^+^ exchange inhibitor cariporide, dominant-negative soluble PDGFR-β and selective PDGFR tyrosine kinase inhibitor AG1295 have been confirmed to reduce HSCs proliferation and attenuate hepatic fibrosis (13–19). We have previously developed an anti-PDGFR-β ribozyme to block the signal transduction pathway of PDGF, inhibit HSCs proliferation and activation and decrease the production of ECM subsequently *in vitro* (20). Here, we demonstrated that downregulation of PDGFR-β by RNA interference (RNAi) could also result in the inhibition of ERK phosphorylation; thus, the blockage of the MAPK signal transduction pathway and the suppression of HSCs proliferation *in vitro*, the liver injury and hepatic fibrosis induced by DMN and BDL could also be relieved by PDGFR-β shRNA treatment. In addition, we confirmed the reduced α-SMA expression by PDGFR-β shRNA treatment not only *in vitro* but also *in vivo*, suggesting that the activation of HSCs was suppressed by PDGFR-β shRNA. This fits well with other studies demonstrating that inhibitors of PDGF decreased α-SMA immunoreactivity (21).

RNA interference is the process of sequence-specific, post-transcriptional gene silencing, initiated by double-stranded RNA. This is a multistep process that involves generation of 19–21 nucleotide siRNA, resulting in degradation of the homologous RNA (22). Delivery of chemically synthesized siRNAs can result in sequence-specific translation arrest *in vitro*. However, the disadvantages of chemically synthesized siRNA, such as a higher cost, increased synthesis time, easy degradation by serum nucleases and transient downregulation of gene expression, restrict its application *in vivo* (23). To overcome these problems, vector-based systems expressing shRNA that are cleaved by the endogenous nuclease Dicer to produce siRNA have been developed (24). The shRNA was structurally made by stem–loop type of siRNA. In this study, we constructed a PDGFR-β shRNA expression plasmid, in which PDGFR-β shRNA embedded in an miR30-based context was driven by a CMV promoter. Our results clearly demonstrated the antifibrogenic effect of PDGFR-β shRNA expression plasmid on an experimental hepatic fibrosis animal model induced by BDL and DMN.

A number of different methods have been developed to deliver siRNA *in vivo*. Among the methods attempted in rodents, rapid infusion by hydrodynamic injection of siRNA via the tail vein has been recommended as one of the best techniques to deliver siRNA into the liver (25–27). Several groups have used this technique to introduce siRNA- or shRNA-expressing constructs successfully into mice, achieving efficient delivery and subsequent silencing of target genes in the liver (28, 29). In this study, we also used this method to deliver the plasmid-expressing PDGFR-β shRNA into the target organ: the liver. What concerned us is whether hydrodynamic injection can deliver siRNA into HSCs, especially activated HSCs. We detected the LacZ reporter gene expression in BDL rats and DMN rats by double-label immunofluorescence, and the results demonstrated that the LacZ gene can be expressed in not only hepatocytes but also α-SMA-positive HSCs. Because PDGFR-β is only expressed in HSCs in the liver, we deduce that PDGFR-β shRNA driven by the CMV promoter can only exert its effect in HSCs, although it can be delivered into different cell types including hepatocytes. As the highest gene-silencing efficacy of PDGFR-β shRNA appeared in 72 h *in vitro*, we delivered the plasmid into rats by a hydrodynamics-based transfection method every 5 days to achieve stable expression of PDGFR-β siRNA *in vivo*. Considering the poor conditions of rats after BDL operation and DMN treatment, we decreased the injection volume to 10 ml/200 g body weight from the second injection.

The lessened liver injury by PDGFR-β shRNA treatment was documented by our study, which was characterized by the lower serum levels of aminotransferase and bilirubin in PDGFR-β shRNA treatment rats than in the control rats. The results are interesting because the increased serum aminotransferase reflects the damage of hepatocytes, while the target cell of PDGFR-β shRNA is HSCs. Friedman (30) believed that fibrosis in the Disse space may block the exchange of molecules between the sinusoidal space and the hepatocytes, thereby impairing the functioning of hepatocytes. Thus, a reduction of fibrosis in this space might improve liver function more than might otherwise be expected. Moreover, another study eliminated transforming growth factor-β (TGF-β) signalling by adenovirus-mediated expression of a dominant-negative type-II TGF-β receptor in a DMN-induced hepatic fibrosis rat model, and the results revealed that it could reduce ALT levels of the model control more than 60-fold, and the rats in the model control all died of liver dysfunction (31). Therefore, the improved liver function by PDGFR-β shRNA may be attributed to the decreased ECM deposition.

In conclusion, the data presented here provide evidence that PDGFR-β shRNA is active as an antifibrogenic gene therapeutic method able to reduce the expression of PDGFR-β protein and inhibit the activation of HSCs in fibrotic liver. The beneficial effects of RNAi technique make it a promising therapeutic method for the treatment of hepatic fibrosis in the future.

## Abbreviations

α-SMA: α-smooth muscle actin
ALT: alanine aminotransferase
AST: aspartate aminotransferase
BDL: bile duct ligation
DB: direct bilirubin
DMN: dimethylnitrosamine
ECM: extracellular matrix
ERK: extracellular-signal-related kinase
HSCs: hepatic stellate cells
MAPK: mitogen-activated protein kinases
PDGF: platelet-derived growth factor
PDGFR-β: PDGF receptor β subunit
RNAi: RNA interference
siRNA: small interference RNA
shRNA: short hairpin RNA
TB: total bilirubin
